# Technical outcomes of robotic-assisted surgery versus laparoscopic surgery for rectal tumors: a single-center safety and feasibility study

**DOI:** 10.1007/s00595-023-02758-x

**Published:** 2023-11-01

**Authors:** Jesse Y. Tajima, Ryoma Yokoi, Shigeru Kiyama, Takao Takahashi, Hirokata Hayashi, Toshiya Higashi, Masahiro Fukada, Ryuichi Asai, Yuta Sato, Itaru Yasufuku, Yoshihiro Tanaka, Naoki Okumura, Katsutoshi Murase, Takuma Ishihara, Nobuhisa Matsuhashi

**Affiliations:** 1 Department of Gastroenterological Surgery, Gifu University Hospital, 1-1 Yanagido, Gifu, 501-1194 Japan; 2 Innovative and Clinical Research Promotion Center, Gifu University Hospital, Gifu, Japan

**Keywords:** Rectal tumor, Robot-assisted surgery, Postoperative complications

## Abstract

**Purpose:**

Robot-assisted surgery has a multi-joint function, which improves manipulation of the deep pelvic region and contributes significantly to perioperative safety. However, the superiority of robot-assisted surgery to laparoscopic surgery remains controversial. This study compared the short-term outcomes of laparoscopic and robot-assisted surgery for rectal tumors.

**Methods:**

This single-center, retrospective study included 273 patients with rectal tumors who underwent surgery with anastomosis between 2017 and 2021. In total, 169 patients underwent laparoscopic surgery (Lap group), and 104 underwent robot-assisted surgery (Robot group). Postoperative complications were compared via propensity score matching based on inverse probability of treatment weighting (IPTW).

**Results:**

The postoperative complication rates based on the Clavien–Dindo classification (Lap vs. Robot group) were as follows: grade ≥ II, 29.0% vs. 19.2%; grade ≥ III, 10.7% vs. 5.8%; anastomotic leakage (AL), 6.5% vs. 4.8%; and urinary dysfunction (UD), 12.1% vs. 3.8%. After adjusting for the IPTW method, although AL rates did not differ significantly between groups, postoperative complications of both grade ≥ II (odds ratio [OR] 0.66, 95% confidence interval [CI] 0.50–0.87, *p* < 0.01) and grade ≥ III (OR 0.29, 95% CI 0.16–0.53, *p* < 0.01) were significantly less frequent in the Robot group than in the Lap group. Furthermore, urinary dysfunction also tended to be less frequent in the Robot group than in the Lap group (OR 0.62, 95% CI 0.38–1.00; *p* = 0.05).

**Conclusion:**

Robot-assisted surgery for rectal tumors provides better short-term outcomes than laparoscopic surgery, supporting its use as a safer approach.

**Supplementary Information:**

The online version contains supplementary material available at 10.1007/s00595-023-02758-x.

## Introduction

Colorectal cancer is a major disease that affects 19,000 people annually, ranking third among males, second among females, and third in terms of the overall number of deaths worldwide [1]. Colorectal surgery, which is performed via laparotomy, has shifted to laparoscopic and robot-assisted surgery with an emphasis on minimally invasive procedures. Rectal cancer surgery is particularly challenging among colorectal surgeries because it necessitates dissection in the narrow pelvic cavity along a more complicated anatomical layer, while preserving the urogenital organs and their associated autonomic nervous systems.

Conventional laparoscopic rectal surgery is difficult because the limited flexibility of forceps prevents their straight-line entry into the narrow pelvic cavity. Two recent multicenter randomized controlled trials revealed that laparoscopic surgery for rectal cancer has a higher rate of positive surgical resection margins than open surgery [2, 3]. Robot-assisted surgery for rectal tumors is expected to be useful not only in oncological practice but also in terms of postoperative clinical course and anal functional preservation, as it enables precise surgical manipulation within the narrow pelvic cavity with its multi-joint function and three-dimensional (3D) visualization, in addition to the magnification effect of laparoscopic surgery.

Weber et al. first reported robot-assisted colon resection for benign diseases in 2001, and Pigazzi et al. first reported robot-assisted total mesorectal excision for rectal cancer in 2006 [4, 5]. Since then, the use of robot-assisted rectal surgery has spread rapidly, with the total number of surgeries using the da Vinci Surgical System^®^ reaching more than 800,000 in 2020. The number of robot-assisted rectal surgeries has dramatically increased in Japan since insurance coverage began in 2018.

Robot-assisted surgery for rectal cancer, particularly in lower cases, is expected to reduce the number of surgical wounds, improve oncological outcomes, accelerate postoperative recovery, and reduce postoperative complications. Wang et al. reported a meta-analysis of the outcomes of robot-assisted and laparoscopic surgeries for rectal cancer and identified lower complication rates with robot-assisted surgery [6]. However, their report also mentioned longer operative periods, and included many reports that did not determine the superiority of robot-assisted surgery. Thus, the advantages of robot-assisted surgery for minimally invasive rectal cancer remain unclear.

Therefore, in this study, we compared the short-term outcomes of laparoscopic and robot-assisted surgery for rectal tumors to verify the safety of the robot-assisted approach.

## Patients and methods

### Patients and study design

This was a single-center, retrospective study. All procedures and perioperative management were performed at Gifu University Hospital. Robot-assisted surgeries were performed using the da Vinci robotic system (Intuitive Surgical, Sunnyvale, CA, USA), and all surgeries were performed by four surgeons certified by the National Society for Endoscopic Surgery in Japan. Patients who underwent laparoscopic or robot-assisted resection of rectal tumors between 2017 and 2021 were selected, and those who did not undergo anastomosis were excluded. The patients were divided into a laparoscopic surgery group (Lap group) and robot-assisted surgery group (Robot group).

This study was approved by the Central Ethics Committee of Gifu University (Approval Number: 2019–147).

### Outcomes of interest

The following outcomes and parameters were used to compare the two operative approaches. The primary outcome of this study was postoperative complication rates, assessed as Clavien-Dindo grade ≥ II, grade ≥ III, anastomotic leakage (AL), and urinary dysfunction of all grades. The age, sex, body mass index (BMI), American Society of Anesthesiologists physical status (ASA-PS), diabetes mellitus (DM), prognostic nutritional index (PNI), lateral pelvic lymph node (LPL) dissection, tumor size, cT and cN factors, diverting stoma, and preoperative treatment were incorporated as propensity score factors.

### Extracted data

The following clinical data were extracted: age, sex, physical status (PS), BMI, presence of DM, and PNI scores as the patient’s clinical background, histopathological findings, tumor size, cTNM stage, and details of preoperative treatment as the oncological factors and operative period, operative blood loss, presence of LPL dissection, number of dissected lymph nodes, presence of diverting stoma, first postoperative bowel movement, postoperative complications, and length of postoperative hospital stay as the perioperative factors.

### Definition of each parameter

The PS was categorized based on the ASA-PS [7]. The PNI is a nutritional indicator calculated using the formula (10 × albumin) + (0.005 × total lymphocytes) [8]. cT, cN, and cM stages were classified according to the eighth edition of the Union for International Cancer Control.

Postoperative complications were evaluated as the endpoint and classified according to the Clavien-Dindo classification [9], which categorizes surgical complications from grades I to V based on the invasiveness of the necessary treatment. Grade I entails no treatment or wound infection at the bedside; grade II entails medical therapy; grade IIIa entails surgical, endoscopic, or radiological intervention, but not general anesthesia; grade IIIb entails general anesthesia; grade IV entails life-threatening complications that require intensive care; and grade V entails patient death. We retrospectively reviewed the patient records to determine the incidence of complications during hospitalization and within 30 days of surgery. Serious complications were defined as those of grade ≥ IIIa. Mortality (grade V) was defined as in-hospital postoperative mortality from any cause. Postoperative AL, a major complication of rectal resection, was also evaluated, regardless of the Clavien-Dindo grade.

### Statistical analyses

The inverse probability of the treatment weighting method was used to adjust for baseline differences between the lap and robot groups, and the inverse probability of treatment weighting (IPTW) method was used. A multivariate logistic regression model based on the age, sex, BMI, ASA-PS, DM, PNI, LPL dissection, tumor size, cT and cN factors, diverting stoma, and preoperative treatment was used to estimate the propensity score of each patient. The missing values for all explanatory variables were imputed using a multiple imputation method, generating five sets of imputed datasets based on the aregImpute (www.rdocumentation.org/packages/Hmisc/versions/4.4-2/topics/aregImpute). The coefficients of the logistic regression model obtained from all five datasets were pooled using Rubin’s rule. Patient characteristics were compared before and after weighting by the inverse of the propensity score using the standardized mean difference (SMD), and the groups were considered homogeneous for each variable when the SMD was ≤ 0.2. To determine differences in the Clavien-Dindo grade and leakage between the two groups, we used a multivariable logistic regression model with weighting of each observation using IPTW. The variance of the coefficients was estimated using the Huber-White sandwich estimator to consider data clustering by weighting.

All statistical analyses were performed with a two-sided significance level of 5% using the R software version 4.2.2 (www.r-project.org).

## Results

### Patient characteristics

**Fig. 1:**
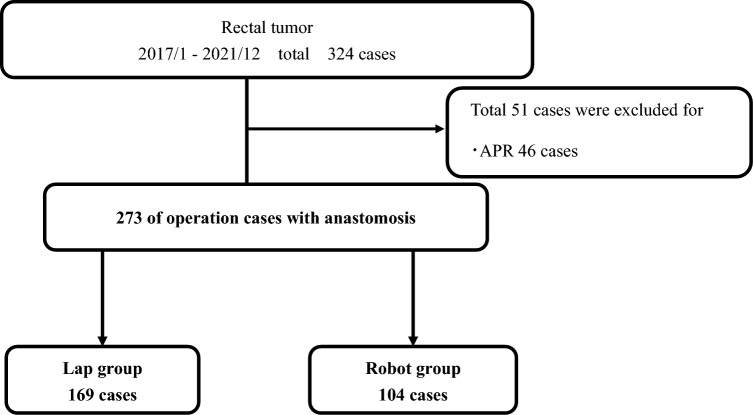
Flowchart of the study

The patient selection scheme is illustrated in Fig. 1. In total, 324 rectal tumors were resected laparoscopically or with robot assistance between 2017 and 2021. Of these patients, 273 who had undergone anastomosis were retrospectively analyzed. The total patient background and oncological factors are shown in Table 1, and perioperative factors are shown in Table 2. A total of 67% were male, and the median age was 67 years old. Among these, 10% underwent preoperative treatment with neoadjuvant chemoradiotherapy and/or neoadjuvant chemotherapy. Regarding the surgical approach, 169 patients (62%) underwent the laparoscopic approach, and 104 (38%) underwent the robot-assisted approach. Diverting stomas were created in 122 (45%) patients. Postoperative complications were Clavien-Dindo grade ≥ II in 70 patients (26%) and grade ≥ III in 25 (9%). AL occurred in 16 (5.9%) patients, and urinary dysfunction occurred in 18 (6.6%).

**Table 1:** Patients’ background characteristics and oncological factors

	Lap (*N* = 169)		Robot (*N* = 104)	*p*-value†
Gender (Male, %)	115 (68.0)		67 (64.4)	0.63
Age (year)^a^	65.0 ± 11.7		64.8 ± 10.7	0.87
BMI (kg/m^2^)^a^	23.4 ± 4.45		23.0 ± 3.05	0.30
ASA-PS (%)				
1	48 (28.4)		31 (29.8)	0.96
2	115 (68.0)		68 (65.4)	
3	6 (3.6)		5 (4.8)	
DM (%)	37 (21.9)		18 (17.3)	0.45
PNI^a^	51.0 ± 5.79		51.8 ± 4.91	0.20
Histology (%)				
Differentiated AC	155 (91.7)		93 (89.4)	0.15
Undifferentiated AC	9 (5.3)		2 (1.9)	
NET	4 (2.4)		5 (4.8)	
Unknown	1 (0.6)		3 (2.9)	
Tumor size (mm)^a^	39.6 ± 20.8		32.1 ± 17.2	** < 0.01**
Distance from AV (cm)^a^	9.9 ± 5.9		9.6 ± 6.0	0.41
cT factor (%)				
1	28 (16.7)		37 (35.9)	** < 0.01**
2	24 (14.3)		26 (25.2)	
3	82 (48.8)		33 (32.0)	
4	34 (20.2)		7 (6.8)	
cN factor (%)				
0	88 (52.1)		76 (73.1)	** < 0.01**
1	54 (32.0)		18 (17.3)	
2	19 (11.2)		4 (3.8)	
3	8 (4.7)		6 (5.8)	
cM factor (%)				
0		150 (88.8)	97 (93.3)	0.22
1		19 (11.2)	7 (6.7)	
Preoperative treatment (%)				
NAC		11 (6.9)	5 (4.8)	0.26
NACRT		4 (2.4)	5 (4.8)	
NAC + NACRT		3 (1.8)	0 (0)	

*p* value and SMD value with statistically significant differences between the two groups are displayed in bold

*SD*: standard deviation, *BMI* body mass index, *ASA-PS* American society of anesthesiologists physical status, *DM* diabetes mellitus, *PNI* prognostic nutritional index = (10 × Alb) + (0.005 × TLC), *AD* adenocarcinoma, *NET* neuroendocrine tumor, *AV* anal verge, *NAC* neoadjuvant chemotherapy, *NACRT* neoadjuvant chemoradiotherapy

^a^Mean ± SD

^b^Clavien-Dindo grade

†Pearson’s chi-squared test

**Table 2:** Perioperative factors

	Lap (*N* = 169)	Robot (*N* = 104)	*p*-value^†^
Operative period (min)^a^	302.4 ± 128.4	290.3 ± 97.8	0.38
Operative blood loss (ml)^a^	48.8 ± 91.8	20.3 ± 33.8	** < 0.01**
LPL dissection (%)	18 (10.7)	5 (4.8)	0.09
Number of lymph nodes collection^a^	19.2 ± 10.6	15.4 ± 8.3	** < 0.01**
Diverting stoma (%)			
Total	73 (43.2)	49 (47.1)	
Ileostomy	31 (18.3)	24 (23.1)	0.53
Colostomy	42 (24.9)	25 (24.0)	0.48
First defecation (day)^a^	3.9 ± 2.6	3.8 ± 1.9	0.66
Postoperative complication (%)^b^			
All grade	61 (43.2)	30 (28.8)	0.22
≥ Grade II	49 (29.0)	20 (19.2)	0.07
≥ Grade III	18 (10.7)	6 (5.8)	0.17
AL	11 (6.5)	5 (4.8)	0.56
UD	14 (12.1)	4 (3.8)	0.15
Postoperative hospital stay (day)^a^	13.0 ± 6.6	14.5 ± 5.8	0.06

*p* value and SMD value with statistically significant differences between the two groups are displayed in bold

*D* standard deviation, *LPL* lateral pelvic lymph node, *AL* anastomotic leakage, *UD* urinary dysfunction

^a^Mean ± SD

^b^Clavien-Dindo grade

^†^Pearson's chi-squared test. S

### Postoperative complications

Details of postoperative complications are shown in Table 3. The most frequent postoperative complication was urinary infection/dysfunction (18 cases), followed by AL (16 cases), abdominal infection (12 cases), ileus (12 cases), and surgical site infection (9 cases). Urinary infection/dysfunction, anastomotic leakage, and ileus were higher in the Lap group than in the Robot group. As all urinary infections were caused by urinary dysfunction, all 18 cases were analyzed as having urinary dysfunction.

**Table 3:** Details of postoperative complications of all grades

	^a^Total (N = 91)	Grade I	Grade II	Grade IIIa	Grade IIIb	Grade IV	Grade V
Urinary infection/Urinary dysfunction	18 (14/4)	0	18	0	0	0	0
Anastomotic leakage	16 (11/5)	0	1	11	3	0	0
Abdominal infection	12 (6/6)	0	8	4	0	0	0
Ileus	12 (9/3)	3	4	5	0	0	0
SSI	9 (4/5)	7	2	0	0	0	0
Chylous ascites	7 (5/2)	5	2	0	0	0	0
Respiratory infection	3 (2/1)	0	2	0	0	0	1
(abdominal/anastomotic) Bleeding	2 (1/1)	1	0	1	0	0	0
High output syndrome	2 (1/1)	2	0	0	0	0	0
Arthritis	2 (2/0)	2	0	0	0	0	0
Lower extremity neuropathy	2 (1/1)	0	2	0	0	0	0
Pseudomembranous enteritis	2 (2/0)	0	2	0	0	0	0
DVT	1 (1/0)	0	1	0	0	0	0
Compartment syndrome	1 (0/1)	0	1	0	0	0	0
Cholecystitis	1 (1/0)	0	1	0	0	0	0
DGE	1 (1/0)	1	0	0	0	0	0
Anastomotic blood flow disorder	1 (1/0)	0	1	0	0	0	0

*SSI* surgical site infection, *DVT* deep vein thrombosis, *DGE* delayed gastric emptying

^a^Total number (lap group/robot group)

### Association between the surgical approach and the short-term outcomes

Comparison of the Lap and Robot groups revealed significant differences in intraoperative blood loss, tumor size, preoperative T and N stages, and the number of lymph nodes dissected (*P* < 0.001, *P *= 0.001, *P* < 0.001, *P* = 0.0025, and *P* = 0.005, respectively). Regarding postoperative complications in the Lap and Robot groups, Clavien-Dindo grade ≥ II was observed in 49 (29.0%) vs. 20 cases (19.2%), grade ≥ III in 18 (10.7%) vs. 6 cases (5.8%), AL (all grades) in 11 (6.5%) vs. 5 cases (4.8%), and urinary dysfunction (all grades) in 14 (12.1%) and 4 cases (3.8%) (Table 2).

The IPTW method revealed no marked difference between the groups with SMD ≤ 0.2 for all variables (Table 4). Although the rates of AL did not differ significantly between groups (odds ratio [OR] 0.76, 95% confidence interval CI 0.46–1.27, *p* = 0.296), postoperative complications of grade ≥ II (OR 0.66, 95% CI 0.50–0.87, *p* = 0.004), and grade ≥ III (OR 0.29, 95% CI 0.16–0.53, *p* < 0.001) were significantly less frequent in the Robot group than in the Lap group, the difference was more remarkable in terms of complications of grade ≥ III. As for urinary dysfunction, although there was no significant difference between the groups, it tended to be less frequent in the Robot group than in the Lap group (OR 0.62, 95% CI 0.38–1.00; *p* = 0.05) (Table 5).

**Table 4:** Patients’ background characteristics and oncological factors before and after IPTW

		Unadjusted			IPTW adjusted		
		Lap (*N* = 169)	Robot (*N* = 104)	SMD	Lap (*N* = 272)	Robot (*N* = 274)	SMD
Gender (Male, %)		115 (68.0)	67 (64.4)	0.077	183 (67.2)	189 (68.9)	0.037
Age (year)^a^		65.0 ± 11.7	64.8 ± 10.7	0.021	64.9 ± 12.0	65.3 ± 10.4	0.036
BMI (kg/m^2^)^a^		23.4 ± 4.4	23.0 ± 3.1	0.124	23.2 ± 4.3	23.1 ± 3.1	0.040
	1	48 (28.4)	31 (29.8)	0.074	82 (30.0)	85 (31.2)	0.027
ASA-PS (%)	2	115 (68.0)	68 (65.4)		181 (66.3)	179 (65.4)	
	3	6 (3.6)	5 (4.8)		10 (3.7)	9 (3.4)	
DM (%)		37 (21.9)	18 (17.3)	0.116	54 (19.8)	54 (19.6)	0.005
PNI^a^		51.0 ± 5.8	51.8 ± 4.9	0.157	51.3 ± 5.8	51.5 ± 4.8	0.037
LPL dissection (%)		18 (10.7)	5 (4.8)	**0.220**	24 (8.7)	26 (12.7)	0.031
Tumor size (mm)^a^		39.6 ± 20.8	32.1 ± 17.2	**0.397**	36.6 ± 20.4	37.1 ± 18.6	0.025
cT factor (%)	1	28 (16.7)	37 (35.9)	**0.671**	66 (24.2)	65 (23.8)	0.025
	2	24 (14.3)	26 (25.2)		49 (18.2)	49.4 (18.1)	
	3	82 (48.8)	33 (32.0)		114 (42.3)	118 (43.4)	
	4	34 (20.2)	7 (6.8)		41 (15.3)	40 (14.6)	
cN factor (%)	0	88 (52.1)	76 (73.1)	**0.496**	163 (59.8)	165 (60.3)	0.047
	1	54 (32.0)	18 (17.3)		73 (26.8)	76 (27.7)	
	2	19 (11.2)	4 (3.8)		23 (8.6)	20 (7.4)	
	3	8 (4.7)	6 (5.8)		13 (4.9)	12 (4.5)	
Preoperative treatment (%)		18 (10.7)	10 (9.6)	0.034	30 (10.8)	31 (11.2)	0.013
Diverting stoma (%)		73 (43.2)	49 (47.1)	0.079	123 (45.2)	123 (44.8)	0.009

*p* value and SMD value with statistically significant differences between the two groups are displayed in bold

*SMD* standardized mean difference, *SD* standard deviation, *BMI* body mass index, *ASA-PS* American Society of Anesthesiologists physical status, *DM* diabetes mellitus, *PNI* prognostic nutritional index = (10 × Alb) + (0.005 × TLC), *LPL* lateral pelvic lymph node

^a^Mean ± SD. IPTW: inverse probability of treatment weighting

**Table 5:** Risk of postoperative complications in the robot group vs. the lap group

Postoperative complication	Odds Ratio	95%CI	*p*-value†
Clavien-Dindo grade			
≥ Grade II	0.66	0.50–0.87	** < 0.01**
≥ Grade III	0.29	0.16–0.53	** < 0.01**
Urinary dysfunction (All grade)	0.62	0.38–1.00	0.05
Anastomotic leakage (All grade)	0.76	0.46–1.27	0.30

*p* value and SMD value with statistically significant differences between the two groups are displayed in bold

After IPTW

*IPTW* inverse probability of treatment weighting, *CI* confidence interval

†Pearson’s chi-squared test

## Discussion

This study found that robot-assisted surgery significantly reduced postoperative complications compared with laparoscopic surgery. In particular, urinary dysfunction, which is one of the most frequent postoperative complications, tended to less frequent in the Robot group than in the Lap group. The novelty of this study lies in its focus on anastomotic leakage and urinary dysfunction, which are clinically important morbidities. Furthermore, this result has a statistical impact when the IPTW method is used to remove various patient background biases.

Although a few studies have demonstrated the safety of robot-assisted surgery for rectal cancer, no strong evidence supports its superiority. Many randomized trials and meta-analyses have reported that robot-assisted surgery is superior to laparoscopy and associated with a significantly lower rate of conversion to laparotomy. Conversely, many reports have identified no significant differences in intraoperative blood loss, postoperative complications, or postoperative hospital stay, whereas some studies have reported inferior outcomes, such as a prolonged operation time [6, 10–15]. In the 2017 ROLARR trial, Jayne et al. conducted a large multicenter randomized controlled trial (RCT) comparing the short-term results of robot-assisted versus laparoscopic surgery for rectal cancer, determining the superiority of robot-assisted surgery over laparoscopic surgery in terms of perioperative outcomes [16], but only in a limited subgroup of obese or male patients. Recently, Feng et al. conducted a large multicenter RCT of more than 1000 patients in 2022. That study reported that the patients in the robotic group had fewer postoperative complications than those in the laparoscopic group (*p* = 0.003) [17]. However, few reports have examined the complications in detail, including large-scale clinical trials.

Techniques and instruments for rectal cancer surgery have undergone major changes in recent years, and endoscopic and robot-assisted surgeries have become widely available. At our institution, since the number of qualified surgeons for endoscopic and robotic surgery has increased over the past five years and surgical procedures have become standardized, comparing data older than five years might have revealed a large bias. Therefore, we deemed a comparison within the most recent five-year period appropriate for analysis.

In this study, using the IPTW method, robot-assisted surgery resulted in significantly fewer postoperative complications of Clavien–Dindo grade ≥ II and ≥ III than those associated with laparoscopic surgery. Furthermore, we examined the major postoperative complications in detail. Although we observed a trend toward lower rates of AL robot-assisted surgery than in laparoscopic surgery, the difference was not significant. Regarding urinary dysfunction, although there was no statistically significant difference between the groups, the rate tended to be even lower than AL in the Robot group. Urinary dysfunction and infection were the most common postoperative complications in our study, being found in 18 cases, suggesting that robot-assisted surgery was significantly associated with a lower rate of these complications than laparoscopic surgery.

Preoperative treatment, especially neoadjuvant chemoradiation therapy (NACRT), is a risk factor for pelvic dysfunction, such as urinary dysfunction, after rectal cancer surgery [18, 19]. Therefore, a sub-analysis excluding the 12 patients with NACRT in this study was conducted, and postoperative complications of urinary dysfunction (OR, 0.54; 95% CI 0.33–0.90; *p* = 0.02) were found to be significantly less frequent in the Robot group than in the Lap group (Supp. Tables 1, 2, 3, 4). These results indicate that the robotic approach does indeed contribute to the prevention of urinary dysfunction.

In rectal cancer resection, the urological function must be preserved even while complete tumor resection is pursued. Postoperative urinary dysfunction is often caused by intraoperative injury to the pelvic visceral nerves or pelvic plexus. Robot-assisted surgery has a more stable high-resolution field of view and multi-joint capability than laparoscopic surgery and enables accurate visualization of the anatomy and a safe approach, which can help preserve the pelvic nerves [20]. Most reports of urogenital dysfunction after rectal cancer surgery used the International Prostate Symptom Score (IPSS) and/or the International Index of Erectile Function Scores (IIEF-5), and many have reported that both the IPSS and IIEF-5 are better in robot-assisted surgery than in laparoscopic surgery [21–25]. Tang et al. reported a significantly faster recovery rate for urinary dysfunction with robot-assisted surgery than with laparoscopic surgery [26].

In the present study, although robot-assisted surgery was associated with a lower incidence of AL than laparoscopic surgery, the incidence was also low in the Lap group (6.5%), suggesting that patient background, improvements and innovations in anastomotic devices, and evaluation of anastomotic blood flow might have contributed more to the improvement than the surgical approach itself. For anastomotic devices, there is some concern that the number of staples used for anastomosis increases the number of small defects between staple lines, which may cause AL. Furthermore, Kim et al. reported in their prospective study that the use of two or more staples in anastomosis was associated with AL, and Fukada et al. reported that the number of staples used for anastomosis was significantly higher in male patients than in females, in procedures performed close to the anal verge than in other procedures, and in patients with a longer operative time than in those with a shorter time [27, 28].

Recently, indocyanine green fluorescence angiography (ICG-FA) has been widely used in colorectal surgery to evaluate the blood flow at anastomotic sites. ICG-FA is a near-infrared fluorescent dye that can be detected by imaging systems. It can be used to detect areas of vascular failure and, if necessary, accurately perform anastomoses in areas with a good blood flow. Some reports have demonstrated the usefulness of ICG-FA in preventing AL during colorectal surgery [29].

Robot-assisted rectal cancer surgery has only been performed for a relatively short period of time thus far, and few studies have reported the long-term outcomes [30]. Various randomized trials and meta-analyses have reported significantly lower circumferential resection margin (CRM) positive rates in robot-assisted surgery than laparoscopic surgery [10, 11], but many others have reported no significant differences in the long-term outcomes [12, 14]. Therefore, the results remain controversial. Establishing the superiority of robot-assisted rectal cancer surgery over laparoscopic surgery in terms of the long-term outcomes will require large-scale randomized controlled trials, such as the ongoing ROLARR or COLRAR trials. Postoperative complications, especially intra-abdominal infections, such as AL, are often reported to be poor long-term prognostic factors. In a meta-analysis, Wang et al. reported that the occurrence of postoperative AL significantly increased the local recurrence rate and was a poor prognostic factor for both the overall and cancer-specific survival [31]. The results suggest that reducing postoperative complications, as in this study, may contribute to prolonging the long-term prognosis.

In addition, in the present study, the number of lymph nodes collected was significantly lower in the Robot group than in the Lap group. One reason for this may be that there was a significant difference in tumor progression between the two groups, which led to a difference in the level of dissection. Second, in Japan, the mesorectal ligament around the tumor is peeled off from the resected specimen, and the lymph nodes are collected. In recent years, to evaluate CRM, the mesentery near the tumor is fixed in formalin without being removed, and in the end, pathologists often count the number of lymph nodes. Having multiple parties handle the resected specimens during our study may have affected the results.

Although this was a single-center study, the surgeons were all certified by the Japan Society for Endoscopic Surgery, a medical advisor always served as the primary surgeon or the first assistant, and the entire team was made up of surgeons who had attended designated training certification sessions. Furthermore, the patients’ background characteristics, oncological factors, and surgical factors, including the surgical equipment used, might have resulted in some biases; however, we believe that such biases were minimized between the two groups due to the implementation of the IPTW method.

One major limitation of this study was that it was not a prospective, randomized study. With the rapid spread of robotic devices, especially in high-volume center hospitals, most rectal tumor surgeries are now performed using robot-assisted approaches; therefore, it is difficult to conduct multicenter randomized controlled trials.

## Conclusion

We identified better short-term outcomes after robot-assisted surgery for rectal tumors than with laparoscopic surgery, suggesting that it is safer than laparoscopic surgery.

### Supplementary Information

Below is the link to the electronic supplementary material.

Supplementary file1 (DOCX 16 KB) Table 1. Patients’ background characteristics and oncological factors after excluding NACRT cases. *Mean ± SD, **Clavien-Dindo grade, †Pearson's chi-squared test. SD: standard deviation, BMI: body mass index, ASA-PS: American Society of Anesthesiologists physical status, DM: diabetes mellitus, PNI: prognostic nutritional index = (10 × Alb) + (0.005 × TLC), AD: adenocarcinoma, NET: neuroendocrine tumor, AV: anal verge, NAC: neoadjuvant chemotherapy, NACRT: neoadjuvant chemoradiotherapy

Supplementary file2 (DOCX 15 KB) Table 2. Perioperative factors after excluding NACRT cases. *Mean ± SD, **Clavien-Dindo grade, †Pearson's chi-squared test. SD: standard deviation, LPL: lateral pelvic lymph node, AL: anastomotic leakage, UD: urinary dysfunction

Supplementary file3 (DOCX 17 KB) Table 3. Patients’ background characteristics and oncological factors before and after IPTW after excluding NACRT cases. * Mean ± SD. NACRT: neoadjuvant chemoradiotherapy, IPTW: inverse probability of treatment weighting, SMD: standardized mean difference, SD: standard deviation, BMI: body mass index, ASA-PS: American Society of Anesthesiologists physical status, DM: diabetes mellitus, PNI: prognostic nutritional index = (10 × Alb) + (0.005 × TLC), LPL: lateral pelvic lymph node

Supplementary file4 (DOCX 13 KB) Table 4. Risk of postoperative urinary dysfunction in the Robot group vs. the Lap group after IPTW after excluding NACRT cases. †Pearson's chi-squared test. NACRT: neoadjuvant chemoradiotherapy, IPTW: inverse probability of treatment weighting, CI: confidence interval

## Data Availability

Not applicable.

## Abbreviations

SMD: Standardized mean difference
IPTW: Inverse probability of treatment weighting
LPL: Lateral pelvic lymph node
OR: Odds ratio
CI: Confidence interval
PS: Physical status
DM: Diabetes mellitus
PNI: Prognostic nutritional index
ASA: American Society of Anesthesiologists
AL: Anastomotic leakage
RCT: Randomized controlled trial
ICG-FA: Indocyanine green-fluorescence angiography
IPSS: International prostate symptom score
IIEF-5: International index of erectile function score
NAC: Neoadjuvant chemotherapy
NACRT: Neoadjuvant chemoradiotherapy
CRM: Circumferential resection margin
