# Supplementary material for: Technical outcomes of robotic-assisted surgery versus laparoscopic surgery for rectal tumors: a single-center safety and feasibility study
Source: Surg Today. 2023 Nov 1;54(5):478–86. doi: 10.1007/s00595-023-02758-x (PMC11026191; doi:10.1007/s00595-023-02758-x)
Supplement: Supplementary file 1 — Supplementary file1 (DOCX 16 KB) Table 1. Patients’ background characteristics and oncological factors after excluding NACRT cases. *Mean ± SD, **Clavien-Dindo grade, †Pearson's chi-squared test. SD: standard deviation, BMI: body mass index, ASA-PS: American Society of Anesthesiologists physical status, DM: diabetes mellitus, PNI: prognostic nutritional index = (10 × Alb) + (0.005 × TLC), AD: adenocarcinoma, NET: neuroendocrine tumor, AV: anal verge, NAC: neoadjuvant chemotherapy, NACRT: neoadjuvant chemoradiotherapy [file 595_2023_2758_MOESM1_ESM.docx]

**Supplementary Table.1**

|  | | |  | Lap (N=162) | Robot (N=99) | p-value† |
| --- | --- | --- | --- | --- | --- | --- |
| Gender (Male, %) | | | | 108 (66.7) | 63 (63.6) | 0.62 |
| Age (year)* | | | | 65.1 ± 11.8 | 64.6 ± 10.8 | 0.76 |
| BMI (kg/m2)* | | | | 23.4 ± 4.5 | 23.0 ± 3.0 | 0.38 |
|  | 1 | | | 46 (28.4) | 31 (31.3) | 0.74 |
| ASA-PS (%) | 2 | | | 110 (67.9) | 63 (63.6) |  |
|  | 3 | | | 6 (3.7) | 5 (5.1) |  |
| DM(%) | | | | 34 (21.0) | 16 (16.2) | 0.31 |
| PNI* | | | | 51.0 ± 5.7 | 51.9 ± 4.8 | 0.16 |
| Histology (%) | Differentiated AC | | | 148 (91.4) | 90 (90.9) | 0.25 |
|  | Undifferentiated AC | | | 9 (5.6) | 2 (2.0) |  |
|  | NET | | | 4 (2.5) | 5 (5.1) |  |
|  | Unknown | | | 1 (0.6) | 2 (2.0) |  |
| Tumor size (mm)* | | | | 40.0 ± 20.7 | 30.0 ± 17.3 | **<0.01** |
| Distance from AV (cm)* | | | | 10.0 ± 6.1 | 15.0 ± 8.4 | 0.37 |
| cT factor (%) | | 1 | | 27 (16.8) | 37 (37.8) | **<0.01** |
|  |  | 2 | | 24 (14.9) | 25 (25.5) |  |
|  |  | 3 | | 78 (48.4) | 30 (30.6) |  |
|  |  | 4 | | 32 (19.9) | 6 (6.1) |  |
| cN factor (%) | | 0 | | 84 (51.9) | 74 (74.7) | **<0.01** |
|  |  | 1 | | 52 (32.1) | 15 (15.2) |  |
|  |  | 2 | | 18 (11.1) | 4 (4.0) |  |
|  |  | 3 | | 8 (4.9) | 6 (6.1) |  |
| cM factor (%) | | 0 | | 147 (90.7) | 92 (92.9) | 0.54 |
|  |  | 1 | | 15 (9.3) | 7 (7.1) |  |
| Preoperative   treatment (%) | | NAC | | 11 (6.8) | 5 (5.1) | 0.57 |
