# Supplementary material for: Technical outcomes of robotic-assisted surgery versus laparoscopic surgery for rectal tumors: a single-center safety and feasibility study
Source: Surg Today. 2023 Nov 1;54(5):478–86. doi: 10.1007/s00595-023-02758-x (PMC11026191; doi:10.1007/s00595-023-02758-x)
Supplement: Supplementary file 2 — Supplementary file2 (DOCX 15 KB) Table 2. Perioperative factors after excluding NACRT cases. *Mean ± SD, **Clavien-Dindo grade, †Pearson's chi-squared test. SD: standard deviation, LPL: lateral pelvic lymph node, AL: anastomotic leakage, UD: urinary dysfunction [file 595_2023_2758_MOESM2_ESM.docx]

**Supplementary Table.2**

|  | |  | Lap (N=162) | Robot (N=99) | p-value† |
| --- | --- | --- | --- | --- | --- |
| Operative period (min)* | | | 261.5 ± 127.4 | 278.0 ± 97.4 | 0.42 |
| Operative blood loss (ml)* | | | 10.0 ± 90.9 | 10.0 ± 32.0 | **<0.01** |
| LPL dissection (%) | | | 16 (9.9) | 5 (5.1) | 0.16 |
| Number of lymph  nodes collection * | | | 17 ± 10.6 | 15.4 ± 8.3 | **<0.01** |
| Diverting stoma (%) | Total | | 66 (40.7) | 44 (44.4) | 0.56 |
|  | Ileostomy | | 30 (18.5) | 23 (23.2) | 0.48 |
|  | Colostomy | | 36 (22.2) | 21 (21.2) |  |
| First defecation (day)* | | | 4.0 ± 2.5 | 4.0 ± 2.0 | 0.77 |
| Postoperative complication (%)** | All grade | | 61 (37.7) | 30 (30.3) | 0.23 |
|  | ≥Grade II | | 48 (29.6) | 19 (19.2) | 0.06 |
|  | ≥Grade III | | 18 (11.1) | 6 (6.1) | 0.17 |
|  | AL | | 10 (6.2) | 5 (5.0) | 0.71 |
|  | UD | | 13 (8.0) | 3 (3.0) | 0.10 |
| Postoperative hospital stay (day)* | | | 13.0 ± 7.2 | 12 ± 5.9 | 0.07 |
