# Supplementary material for: Technical outcomes of robotic-assisted surgery versus laparoscopic surgery for rectal tumors: a single-center safety and feasibility study
Source: Surg Today. 2023 Nov 1;54(5):478–86. doi: 10.1007/s00595-023-02758-x (PMC11026191; doi:10.1007/s00595-023-02758-x)
Supplement: Supplementary file 3 — Supplementary file3 (DOCX 17 KB) Table 3. Patients’ background characteristics and oncological factors before and after IPTW after excluding NACRT cases. * Mean ± SD. NACRT: neoadjuvant chemoradiotherapy, IPTW: inverse probability of treatment weighting, SMD: standardized mean difference, SD: standard deviation, BMI: body mass index, ASA-PS: American Society of Anesthesiologists physical status, DM: diabetes mellitus, PNI: prognostic nutritional index = (10 × Alb) + (0.005 × TLC), LPL: lateral pelvic lymph node [file 595_2023_2758_MOESM3_ESM.docx]

**Supplementary Table.3**

|  |  | **Unadjusted** | | |  | **IPTW adjusted** | | |
| --- | --- | --- | --- | --- | --- | --- | --- | --- |
|  |  | **Lap (N=162)** | **Robot (N=99)** | **SMD** |  | **Lap (N=260)** | **Robot (N=261)** | **SMD** |
| Gender (Male, %) | | 108 (66.7) | 63 (63.6) | 0.064 |  | 171 (65.7) | 175 (67.0) | 0.027 |
| Age (year)* | | 65.1 ± 11.8 | 64.6 ± 10.8 | 0.039 |  | 64.9 ± 12.0 | 65.1 ± 10.2 | 0.023 |
| BMI (kg/m2)* | | 23.4 ± 4.5 | 23.0 ± 3.0 | 0.107 |  | 23.3± 4.3 | 23.1 ± 3.0 | 0.043 |
|  | 1 | 46 (28.4) | 31 (31.3) | 0.099 |  | 79 (30.2) | 86 (32.9) | 0.059 |
| ASA-PS (%) | 2 | 110 (67.9) | 63 (63.6) |  |  | 172 (66.0) | 166 (63.6) |  |
|  | 3 | 6 (3.7) | 5 (5.1) |  |  | 10 (3.8) | 9 (3.5) |  |
| DM (%) | | 34 (21.0) | 16 (16.2) | 0.124 |  | 49 (18.8) | 46 (17.5) | 0.033 |
| PNI* | | 51.0 ± 5.7 | 51.9 ± 4.8 | 0.175 |  | 51.4± 5.7 | 51.6 ± 4.8 | 0.048 |
| LPL dissection (%) | | 16 (9.9) | 5 (5.1) | 0.184 |  | 22 (8.5) | 25 (9.7) | 0.042 |
| Tumor size (mm)* | | 39.6 ± 20.9 | 31.9 ± 17.4 | **0.404** |  | 36.9 ± 20.6 | 37.2 ± 18.8 | 0.014 |
| cT factor (%) | 1 | 27 (16.8) | 37 (37.8) | **0.706** |  | 63 (24.3) | 63 (24.3) | 0.029 |
|  | 2 | 24 (14.9) | 25 (25.5) |  |  | 49 (19.0) | 49 (19.0) |  |
|  | 3 | 78 (48.4) | 30 (30.6) |  |  | 108 (41.9) | 111 (42.9) |  |
|  | 4 | 32 (19.9) | 6 (6.1) |  |  | 38 (14.8) | 36 (13.8) |  |
| cN facrto (%) | 0 | 84 (51.9) | 74 (74.7) | **0.496** |  | 156 (60.1) | 159 (61.0) | 0.055 |
|  | 1 | 52 (32.1) | 15 (15.2) |  |  | 68 (26.2) | 71 (27.0) |  |
|  | 2 | 18 (11.1) | 4 (4.0) |  |  | 22 (8.6) | 19 (7.3) |  |
|  | 3 | 8 (4.9) | 6 (6.1) |  |  | 13 (5.2) | 12.2 (4.7) |  |
| Preoperative treatment (%) | | 11 (6.8) | 5 (5.1) | 0.074 |  | 16 (6.3) | 37 (6.4) | 0.005 |
| Diverting stoma (%) | | 66 (40.7) | 49 (44.4) | 0.075 |  | 110 (42.5) | 108 (41.4) | 0.022 |
