# Supplementary material for: Technical outcomes of robotic-assisted surgery versus laparoscopic surgery for rectal tumors: a single-center safety and feasibility study
Source: Surg Today. 2023 Nov 1;54(5):478–86. doi: 10.1007/s00595-023-02758-x (PMC11026191; doi:10.1007/s00595-023-02758-x)
Supplement: Supplementary file 4 — Supplementary file4 (DOCX 13 KB) Table 4. Risk of postoperative urinary dysfunction in the Robot group vs. the Lap group after IPTW after excluding NACRT cases. †Pearson's chi-squared test. NACRT: neoadjuvant chemoradiotherapy, IPTW: inverse probability of treatment weighting, CI: confidence interval [file 595_2023_2758_MOESM4_ESM.docx]

**Supplementary Table.4**

| **Postoperative complication** | **Odds Ratio** | **95%CI** | **p-value**† |
| --- | --- | --- | --- |
| Urinary dysfunction (All grade) | 0.54 | 0.33 – 0.9 | **0.02** |
